# Impact of Climatic Conditions on the Resveratrol Concentration in Blend of *Vitis vinifera* L. cvs. Barbera and Croatina Grape Wines

**DOI:** 10.3390/molecules26020401

**Published:** 2021-01-14

**Authors:** Gabriele Rocchetti, Federico Ferrari, Marco Trevisan, Luigi Bavaresco

**Affiliations:** 1 Department for Sustainable Food Process, Università Cattolica del Sacro Cuore, Via Emilia Parmense 84, 29122 Piacenza, Italy; gabriele.rocchetti@unicatt.it (G.R.); marco.trevisan@unicatt.it (M.T.); 2 Aeiforia S.r.l, 29027 Piacenza, Italy; federico.ferrari@aeiforia.it; 3 Department of Sustainable Crop Production, Università Cattolica del Sacro Cuore, Via Emilia Parmense 84, 29122 Piacenza, Italy

**Keywords:** stilbenes, polyphenols, wine quality, climatic conditions, terroir

## Abstract

The aim of this work was to investigate the effect of meteorological conditions on resveratrol concentration of red wines produced in Piacenza viticultural region (Italy). In this regard, six representative estates producing Colli Piacentini Gutturnio DOC (a blend of *V. vinifera* L. cvs. Barbera and Croatina) vintage wines were analysed for trans- and cis-resveratrol over an 8-year period (1998–2005). Grapes were taken from the same vineyard in each estate by using the same enological practices over the entire investigated period. The meteorological conditions corresponding to the production areas were recorded, and bioclimatic indices were calculated as well. Overall, cis-resveratrol concentration was negatively correlated to Huglin index and August mean temperature, whilst positive correlation coefficients were found when considering the Selianinov index and the rainfall of September.

## 1. Introduction

Resveratrol is a naturally occurring phytoalexin that is produced by several plants in response to injury [1]. It exerts multiple biological activities, including anti-inflammatory, antiproliferative, and antioxidant effects [2]. Structurally, this compound is a stilbenoid that was first isolated in 1939 from the roots of the white hellebore (*Veratrum grandiflorum*) and presumably received its name because of its analogy with the benzene-1,3-diol resorcinol and being isolated from the Veratrum species. Subsequently, resveratrol was isolated from several other plants, fruits, and derivatives, from grapes, wines, apples, raspberries, blueberries, pistachios, plums, peanuts, and a multitude of medicinal and edible plant species undergoing response to stress conditions [3]. Experimental and preclinical studies have shown that resveratrol has several health-promoting properties, including cardioprotective effects, chemo-preventive activity in diverse cancers, and a capacity to extend the lifespan of lower organisms [4,5,6].

Regarding the biosynthetic pathway, stilbenes are produced by the phenylpropanoid pathway; stilbene synthase (StSy) is the key enzyme, and it produces resveratrol, the basic monomer, which can be glycosylated, hydroxylated, methylated or converted into more complex compounds [7]. Interestingly, as previously reviewed [8], it is known that in grapevine, under the same biotic and/or abiotic elicitation conditions, tissue levels of resveratrol (and its glucoside derivatives—piceids) can be affected by the grape variety, the clone, the meteorological conditions, the soil type, and by viticulture practices [9]. Also, the production of wines containing high levels of resveratrol (>2 mg/L) depends on quality-oriented viticulture (suitable terroirs and sustainable viticulture practices) and winemaking technologies that prevent degradation of the compound [10,11]. Also, for the last 15 years, research has been conducted to study varieties of grapevine, wine, and grape juices using different extraction methods and solvents, to find a methodology leading to extracts with maximum yield of bioactive compounds, such as resveratrol [12].

In previous studies, different analytical methods have been used to detect and quantify resveratrol in red wine. Montsko et al. [13] analysed 42 red wines from Hungary to determine trans-resveratrol concentration, whilst Rastija et al. [14] analysed the resveratrol content of 12 red wines from Croatia, thus highlighting the correlations between the stilbene and geographical locations, such as soil properties of the vineyard, sunlight exposure and diurnal temperature. 

Therefore, considering the health-promoting properties of resveratrol and its changes according to different climatic conditions, in this work the quantitative differences of trans- and cis resveratrol (belonging to stilbenes) in six commercial “Colli Piacentini” Gutturnio DOC wines, representing a blend of cvs. Barbera and Croatina, from different growing regions in Piacenza province (i.e., Val Tidone, Val Nure, Val Chiavenna, and Val d’Arda) were evaluated. Wine samples were collected over a period of eight consecutive vintages (i.e., 1998–2005). The aim was to investigate the potential correlations between resveratrol and climatic conditions of eight consecutive vintages of the Colli Piacentini DOC wines. Accordingly, the “Colli Piacentini DOC” is characterized by an area of about 5200 ha and is located in the hilly area of the province of Piacenza (Emilia-Romagna region, Italy).

## 2. Results and Discussion

### 2.1. Evaluation of the Different Bioclimatic Indices Over an Eight-Year- Period

In this study, a survey was conducted to determine the cis- and trans-resveratrol values of red wines obtained from the viticultural area of Piacenza (Italy). The total resveratrol concentration was measured as a result of analyzing wines that were produced using the same vinification process in six different localities, varying in altitude. The bioclimatic indices recorded over a period of eight consecutive vintages (i.e., 1998–2005) are reported in Table 1.

**Figure 1:**
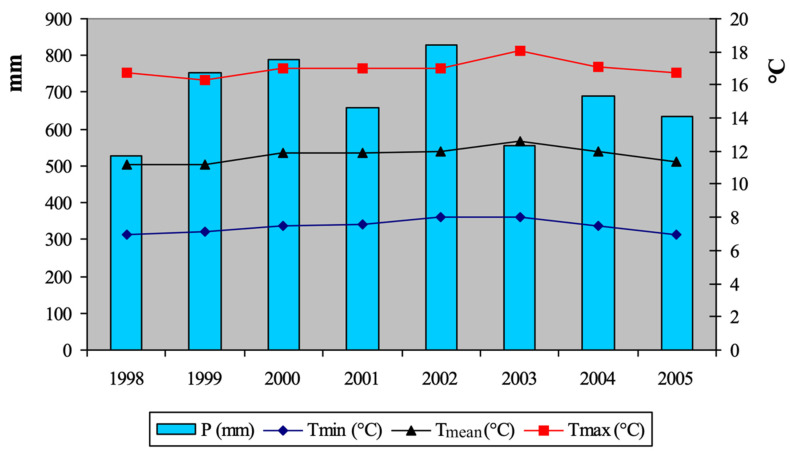
Minimum, mean, maximum temperatures, and mean precipitation of the eight tested vintages: the average values of the six localities are reported.

The trends of some measured meteorological parameters, representative of the entire vineyard area of the” Colli Piacentini DOC” wines, are shown in Figure 1. The hottest year was 2003, i.e., 8.0 °C, 12 °C, and 18.1 °C, minimum, mean and maximum, respectively (annual T and 2673 °C according to IH), while the coolest year was 1997, i.e., 7.1 °C, 11.2 °C, and 16.3 °C, minimum, mean and maximum, respectively, (annual T and 2128 °C according to IH), but with 1998 and 2005 quite similar. The wettest year was 2002 (828 mm), while the driest were 1998 (529 mm) and 2003 (555 mm).

### 2.2. Impact of the Bioclimatic Indices on the Synthesis of Resveratrol

The vintage effect on the concentrations of trans- and cis-resveratrol and total resveratrol is listed in Table 2. Statistically, higher values of total resveratrol (*p* < 0.05) were recorded in 1999 (a relatively cold and wet year in August–September) and in 2005 (similar to the 1999 for temperature values but drier in August–September). The lowest value of trans-resveratrol (0.53 mg/L) was detected in 2000, while the lowest value of cis-resveratrol (0.001 mg/L) was found in 2005 and the lowest value of total resveratrol (0.75 mg/L) corresponded to the 2003 vintage, i.e., the warmest and driest year.

The concentration values of total resveratrol when considering the six different estates over the 8 years are reported in Table 3. Overall, the mean values ranged from 0.84 mg/L found in Gutturnio wine obtained from estate #5 to 1.33 mg/L detected in wines from estate #3; no correlation between resveratrol levels and altitude was found.

**Figure 2:**
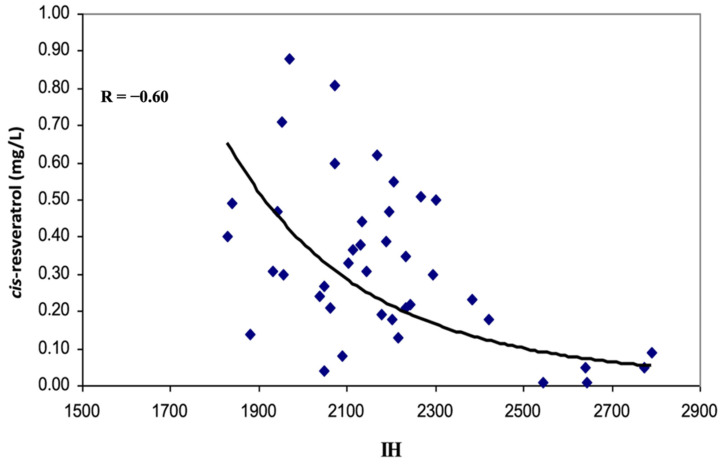
Negative correlations between cis-resveratrol levels and Huglin index (IH).

**Figure 3:**
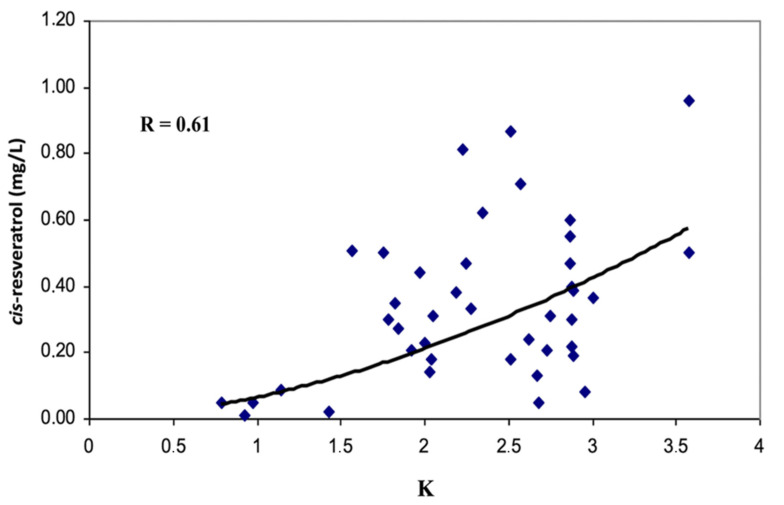
Positive correlations between cis-resveratrol and the Selianinov index (K).

**Figure 4:**
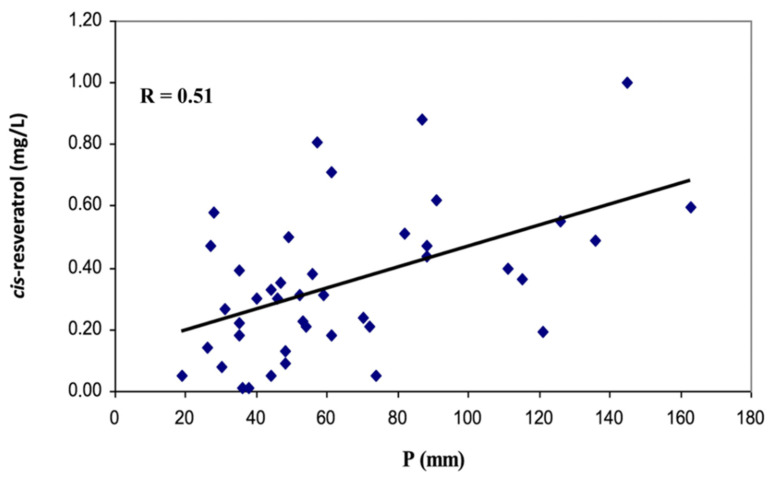
Positive correlations between cis-resveratrol and September precipitation (P).

By correlating the concentrations of resveratrol in Gutturnio wines with the bioclimatic indices, only some regressions were statistically significant, i.e., (1) the negative one between cis-resveratrol and Huglin index (Figure 2), (2) the positive ones between cis-resveratrol and Selianinov index (K), and (3) the positive ones between cis-resveratrol and September precipitation (P) (Figure 3 and Figure 4).

The values of trans-resveratrol were always higher than those of its cis-isomer. Regarding the relationship between the two isomers (trans- and cis-), the related literature reports different results, depending on factors, such as analytical methods [15,16], grape variety and environment [17,18], but the majority of wines had higher concentrations of the trans-isomer than the cis-isomer. Also, the mean concentration of trans-resveratrol found in the wines under investigation (0.89 mg/L) appears to be higher than other values obtained previously on the same wine type (Gutturnio) [19]. The results of this investigation are in the medium to low range concentrations when compared to other grape varieties and environments [20].

Considering the geographical location of the vineyards (latitude) and the specific impact of the climatic conditions, the comparisons with previously published results do not show clear matching. According to Goldberg et al. [17,18], wines produced in cold climates (especially Cabernet Sauvignon) have higher concentrations of resveratrol than wines produced in warm areas, but according to another research by the same group [21] this is not always true. This contradiction can be explained by the interference of other uncontrolled factors, including enological practices. Experimental data on the effect of vineyard elevation on the synthesis of resveratrol in grapes, conducted in Val Tidone (PC), indicate that stilbenes increase up to 300 m. a. s. l. and then decrease at higher altitudes (400 m) [22]. Also, according to literature [23], a major influence on resveratrol (mainly trans-isomer) synthesis is related to *Botrytis cinerea* infection of grapes; however, in this work, the healthy status of the grapes before harvest has not been recorded. Therefore, it is only possible to speculate that the higher the humidity, the higher the *Botrytis* pressure and the corresponding resveratrol synthesis.

In a previous study, Yaman et al. [24], evaluated the impact of vegetation period and climatic conditions on trans-resveratrol concentrations in 21 wine samples (including both Cabernet Sauvignon and Merlot) from different regions in Turkey. The authors reported that resveratrol concentrations in the studied samples varied depending on vegetation period, sunlight duration and mm precipitation in the vineyards. In this regard, the effects of sunlight duration and UV light exposure are known to increase resveratrol concentrations in red wines [25]. The annual precipitation, humidity and temperature affecting the intensity of fungal attacks in the vineyard, can influence the extent of the synthesis of stilbenes [26,27]. In fact, a low incidence of fungal attack (for example gray mold), not visible to the naked eye, is sufficient to trigger the synthesis of stilbenes in the berry, and this occurs under conditions of increasing humidity of between 70 and 80%, during the period of grape maturation [22]. The positive correlation between resveratrol and September precipitation can confirm the link between fungal pressure (favored by humidity) and stilbenes synthesis. According to the literature, the ambient temperature during the grape ripening period also plays an important role, which is negatively correlated with the level of resveratrol in grapes and wine [22,28]. The results of this research partly confirm these findings, referring to the temperatures of the entire growth cycle, as the relations with those of August and September are not significant. The effect of temperature is difficult to explain, because it affects both the physiology of the plant and the fungus population and acts indirectly on the pathogen through the effect on humidity.

## 3. Materials and Methods

### 3.1. Sample Collection

In this work, six different commercial Colli Piacentini Gutturnio DOC wines from eight consecutive vintages (1998–2005), produced by six estates were used. The “Colli Piacentini DOC” represents an area of about 5200 ha and is located in the hilly area of the province of Piacenza (Emilia-Romagna region, Italy). The wines were a blend of *V. vinifera* L. cvs. Barbera and Croatina grapes (55–70% of the former and 30–45% of the latter) produced in representative areas of the entire DOC area (45° Lat N, Table 4). Each estate produced wine during the year with grapes originating from the same vineyard, adopting the same cultivation techniques and using a standardized vinification method. All the estates utilized the same previously reported percentages of Barbera and Croatina grapes each year.

### 3.2. HPLC-DAD Analysis of Cis- and Trans-Resveratrol

The RP-HPLC determination of resveratrol was performed using a liquid chromatography system (Hewlett-Packard 1090 L, Waldbronn, Germany). The system was equipped with an auto-sampler and a DAD. A Phenomenex Luna analytical column (250 × 4.6 mm, 5 μm) with a C18 as a stationary phase was used for compound separation. A gradient mobile phase program was used for compound elution. Mobile phase A consisted of Acetonitrile and mobile phase B consisted of Phosphate Buffer (KH2PO4 + H3PO4; 0.02 M) with a pH of 3. The gradient mobile phase was based on the increase of solvent B from 10% up to 40% in 25 min. The column and the system were equilibrated for 2 min after each analysis run time of 27 min to revert to the starting conditions. The flow rate was 1 mL/min and temperature 20 °C. The injection volume was 10 µL. Compounds in the matrix were quantified using peak areas at 306 and 325 nm. The identification of the compounds in the wines was confirmed by their relative retention times based on available reference standards and UV-visible absorption characteristics. For the quantification step, trans-resveratrol was provided by Sigma-Aldrich (R 5010), whilst cis-resveratrol was obtained by isomerization of trans-resveratrol following UV-exposition (λ = 366 nm) for 40 min, thus reaching a conversion of 90%. The quantification method has been done using the external standard method, in the linear range 0.05–3 mg/L (LOD = 0.05 mg/L). Four replications (*n* = 4) for each wine belonging to the different vintages have been analysed. The instrumental analyses have been done during autumn of the following vintage year.

### 3.3. Evaluation of Bioclimatic Indices

In correspondence with the above-mentioned vineyards, meteorological stations were located in order to record the following parameters: minimum, maximum and average daily temperature (°C), precipitation (mm), and relative humidity (%). These parameters were also used to calculate bioclimatic indices, such as the growing degree days (GDD), the Huglin index (IH), and the Selianinov index (K), as follows: GDD = Σ (daily mean T − 10), (April–September); IH = ((Σ (daily main T − 10) + Σ (daily max T − 10)/2) 1.04 (April–September); K = (rainfall/GDD) 10, (April–September).

### 3.4. Statistical Data Analysis

The quantitative values of the two resveratrol isomers and the total resveratrol (i.e., the sum of both isomers) were statistically analysed by means of the one-way analysis of variance (ANOVA), using as classification criterion a specific vintage. Thereafter, the means were statistically compared with the S-N-K test (*p* < 0.05), in order to evaluate the vintage effect. Finally, to better understand which bioclimatic indices were connected to the quantitative variation of resveratrol in wines, the data were processed by means of regressions aimed at establishing the significant correlations between meteorological parameters and resveratrol in wines.

## 4. Conclusions

In this work, “Colli Piacentini Gutturnio DOC” wines produced from six estates in the period 1998–2005 have been analysed. Each wine was produced during the year with grapes originating from the same vineyard and with the same cultivation techniques, using a standardized vinification method. In this way, it was possible to highlight the vintage as one of the major factors affecting the resveratrol synthesis. It was demonstrated that the concentrations of resveratrol found in “Colli Piacentini Gutturnio DOC” wines, during the period between 1998–2005 varied among vintages (c.v. 33%) but with a tendency to be hindered by hot and dry weather conditions during the grape ripening period. It is important to highlight that, in the current work, only the free resveratrol molecules were investigated, but the compound can be also present in the wines as a glycoside [29]. The link between temperature, humidity, and resveratrol synthesis was also confirmed for this Italian red wine resulting from the significance of the negative correlation between the concentration of cis-resveratrol and the Huglin index, together with the positive correlation detected between the Selianinov index and the precipitation in September.

## Figures and Tables

**Table 1:** Average values of bioclimatic indices and meteorological parameters of the eight vintages.

	1998	1999	2000	2001	2002	2003	2004	2005	Average
GDD (°C) ^1^	1516	1514	1533	1520	1496	1975	1611	1551	1590
IH (°C) ^2^	2143	2128	2199	2170	2097	2673	2250	2158	2228
K ^3^	2.15	2.56	2.59	2.63	2.61	0.96	1.71	2.59	2.23
P (mm) ^4^	320	382	391	385	388	190	274	399	341
P (mm) ^5^	27	113	62	45	112	19	32	71	60
P (mm) ^6^	73	112	48	94	40	36	48	50	63
RH (%) ^7^	66	71	70	68	83	69	59	69	69
RH (%) ^8^	75	78	69	71	81	67	64	80	73
T (°C) ^9^	22.9	21.6	22.2	23.3	20.9	27.2	23.5	20.4	22.8

^1^ Σ (daily mean T − 10), (April–September); ^2^ ((Σ (daily mean T − 10) + Σ (daily max. T − 10)/2) 1.04; ^3^ (P/GDD) 10, (April–September); ^4^ Precipitation (April–September); ^5^ Precipitation (August); ^6^ Precipitation (September); ^7^ Relative Humidity (August); ^8^ Relative Humidity (September); ^9^ mean Temperature (August). Abbreviations: GDD (Growing Degree Days); IH (Huglin Index); K (Selianinov index); P (Precipitations); RH (Relative Humidity).

**Table 2:** Mean values of trans- and cis-resveratrol and total resveratrol content for the different vintages. Values with different superscript letters in the same row are significantly different (*p* < 0.05) as resulting from ANOVA (post-hoc SNK). The average values are also provided.

	1998	1999	2000	2001	2002	2003	2004	2005	Average
trans-resveratrol (mg/L)	0.88 ^ab^	1.21 ^b^	0.53 ^a^	0.75 ^ab^	0.73 ^a^	0.66 ^a^	0.90 ^ab^	1.49 ^b^	0.89
cis-resveratrol (mg/L)	0.58 ^ab^	0.66 ^b^	0.24 ^a^	0.35 ^ab^	0.17 ^a^	0.09 ^a^	0.31 ^ab^	0.001 ^c^	0.30
Total resveratrol (mg/L)	1.46 ^ab^	1.87 ^b^	0.77 ^a^	1.10 ^ab^	0.90 ^a^	0.75 ^a^	1.21 ^ab^	1.49 ^b^	1.19

**Table 3:** Total resveratrol concentrations (mg/L) in the wines from the six estates per year together with the corresponding average values. Mean values with different superscript letters in the same column (^a–h^) and in the same row (^A–F^) are significantly different (*p* < 0.05) for vintage and estate, respectively, as resulting from ANOVA (post-hoc SNK). The average values are also provided.

	Estate	1	2	3	4	5	6	Average
Vintage								
1998		1.16 ^f,B^	1.15 ^d,B^	1.24 ^e,C^	1.92 ^g,E^	1.13 ^e,A^	1.68 ^g,D^	1.46
1999		1.93 ^h,C^	1.49 ^g,A^	2.66 ^h,E^	1.50 ^d,A^	1.57 ^h,B^	2.04 ^h,D^	1.87
2000		0.61 ^b,C^	1.32 ^e,F^	0.82 ^a,E^	0.42 ^a,A^	0.58 ^b,B^	0.72 ^b,D^	0.77
2001		0.65 ^c,A^	1.01 ^b,D^	0.88 ^c,C^	1.51 ^e,F^	0.81 ^d,B^	1.26 ^e,E^	1.10
2002		0.33 ^a,A^	0.63 ^a,B^	1.54 ^f,F^	1.34 ^c,E^	0.64 ^c,C^	1.12 ^c,D^	0.90
2003		0.83 ^e,D^	1.42 ^f,E^	0.84 ^b,D^	0.67 ^b,C^	0.30 ^a,A^	0.48 ^a,B^	0.75
2004		1.38 ^g,D^	1.10 ^c,B^	1.08 ^d,A^	1.79 ^f,E^	1.25 ^f,C^	1.21 ^d,C^	1.21
2005		0.70 ^d,A^	1.65 ^h,D^	1.74 ^g,E^	1.50 ^d,B^	1.46 ^g,B^	1.57 ^f,C^	1.49
**Average**		0.92	1.17	1.33	1.23	0.84	1.22	

**Table 4:** Locality and altitude of each estate providing the red wines under investigation.

Estate	Locality	Altitude (m. a. s. l.)
1	Castell’Arquato (Val d’Arda)	160
2	Carmiano (Val Nure)	280
3	Vigolo Marchese (Val Chiavenna)	150
4	Vicobarone (Val Tidone)	290
5	Vicobarone zone (Val Tidone)	260–300
6	Ziano P.no (Val Tidone)	270

## Data Availability

Not applicable.

## Sample Availability

Not available.
